# Slicing Resource Allocation Based on Dueling DQN for eMBB and URLLC Hybrid Services in Heterogeneous Integrated Networks

**DOI:** 10.3390/s23052518

**Published:** 2023-02-24

**Authors:** Geng Chen, Rui Shao, Fei Shen, Qingtian Zeng

**Affiliations:** 1 College of Electronic and Information Engineering, Shandong University of Science and Technology, Qingdao 266590, China; 2 Shanghai Institute of Microsystem and Information Technology, Chinese Academy of Sciences, Shanghai 200050, China

**Keywords:** 5G/B5G, network slicing, deep reinforcement learning, dueling deep Q network (Dueling DQN), resource allocation and scheduling

## Abstract

In 5G/B5G communication systems, network slicing is utilized to tackle the problem of the allocation of network resources for diverse services with changing demands. We proposed an algorithm that prioritizes the characteristic requirements of two different services and tackles the problem of allocation and scheduling of resources in the hybrid services system with eMBB and URLLC. Firstly, the resource allocation and scheduling are modeled, subject to the rate and delay constraints of both services. Secondly, the purpose of adopting a dueling deep Q network (Dueling DQN) is to approach the formulated non-convex optimization problem innovatively, in which a resource scheduling mechanism and the ϵ-greedy strategy were utilized to select the optimal resource allocation action. Moreover, the reward-clipping mechanism is introduced to enhance the training stability of Dueling DQN. Meanwhile, we choose a suitable bandwidth allocation resolution to increase flexibility in resource allocation. Finally, the simulations indicate that the proposed Dueling DQN algorithm has excellent performance in terms of quality of experience (QoE), spectrum efficiency (SE) and network utility, and the scheduling mechanism makes the performance much more stable. In contrast with Q-learning, DQN as well as Double DQN, the proposed algorithm based on Dueling DQN improves the network utility by 11%, 8% and 2%, respectively.

## 1. Introduction

With the explosive growth of data in mobile networks, 5G mobile communication technologies have matured to meet a wide variety of traffic needs. The two most typical types of services in 5G mobile networks are ultra-reliable and low-latency communication (URLLC) and enhanced mobile broadband (eMBB) [1]. The 5G network provides resources for the two types of users mentioned above in a sliced manner [2,3]. When slicing is performed, the allocation of resources is adjusted by the base station (BS) according to the dynamic demands of user services and adapts to different network states [4]. Slicing of network resources enables data triage management and flexible resource allocation in 5G networks [5,6], and it is also necessary to achieve a high data transmission rate, low latency and high capacity [7,8].

Due to the intense growth of network traffic and the densification of devices, there are multiple problems and great challenges in the allocation and scheduling of resources between different services [9]. For example, in the 5G scenario, when there are users of both eMBB and URLLC service types, it is necessary to allocate a lot of bandwidth resources to users of the eMBB service type within a time slot to ensure that their images and voice have high and stable quality, and it is also necessary to successfully transmit the data packets requested by URLLC service type users within the range of very short delay to meet the characteristics of ultra-high reliability and ultra-low delay [10]. If there is a sudden increase in URLLC traffic in the same area, it will quickly occupy these bandwidth resources to reach its required transmission rate, resulting in an ultra-low latency performance [11]. When bandwidth resources are insufficient, existing works typically prioritize the performance requirements of URLLC by sacrificing the quality of experience (QoE) of eMBB. The rational allocation and scheduling of slicing resources facilitate efficient resource use in hybrid services systems.

In recent years, reinforcement learning (RL) has become a potential solution to the resource allocation problem. The resource allocation algorithm based on RL has improved resource utilization efficiency [12]. As deep reinforcement learning (DRL) has evolved, many research works based on DRL approaches have also been achieved [13,14]. For example, DRL is applied to solve problems with resource allocation [15], network optimization, routing, scheduling and radio control. Chen et al. [16] modeled the problem of auctioning a finite number of channels across scheduling slots to multiple service providers as a stochastic game, then linearly decomposed the Markov decision process for each service provider and derived an online solution based on deep reinforcement learning. In [17], the stochastic decision process in vehicular networking is modeled as a discrete-time single-intelligent Markov decision process (MDP) to address the partial observability and high dimensionality curse of the local network state space faced by each vehicular user device and to make optimal band allocation and group scheduling decisions in a decentralized manner in each scheduling time slot. In [18], a deep Q-network (DQN) algorithm based on discrete normalized advantage functions (DNAF) was studied, and the advantage function was decomposed into two function terms to reduce the computational complexity of the algorithm. In addition, the simulation results verify that the deep Q-learning (DQL) based on the K-nearest neighbor algorithm can converge faster in discrete environments. Sciancalepore et al. [19] proposed the reinforcement learning-based network slice broker (RL-NSB) framework to effectively improve the utilization of the system by considering factors such as traffic flow, mobility, and optimal access control decision. The distributed idea [20] and the effect of randomness noise for spectrum efficiency (SE) and service level agreement (SLA) satisfaction ratio (SSR) are referred to [21]. Hua et al. [21] introduced the generative adversarial network and used it to allocate physical resources among multiple network slices of a single BS, which performs well in terms of demand-aware resource management. Furthermore, Li et al. [22] considered the user mobility based on [19] and utilized the actor–critic based on long short-term memory (LSTM-A2C) algorithm to follow the mobility of users, improving the practicality of the resource allocation system. Yuan et al. [23] provide a DRL-based resource-matching distributed method to maximize energy efficiency (EE) and device-to-device (D2D) capacity through a decentralized approach, match multi-user communication resources to double DQN, and optimize radio channel matching and power allocation. Sun et al. [24] distinguished resource granularity, utilized virtualized coarse resources to obtain provisioning solutions and used fine resources for dynamic slicing, proposing a dueling DQN-based algorithm customized to the diverse needs of users to improve user satisfaction and resource utilization. Chen et al. [25] used an algorithm based on dueling deep Q network (Dueling DQN) combined with bidding for bandwidth resource allocation in two layers to improve the QoE of users and verify the advantages of Dueling DQN over Double DQN in resource allocation. Boateng et al. [26] proposed a new hierarchical framework for autonomous resource slicing in 5G RANs, modeling the seller and buyer pricing and demand problems as a two-stage Stackelberg game to design fair incentives and designing a Dueling DQN scheme to achieve optimal pricing and demand strategies for autonomous resource allocation in negotiated intervals. Zhao et al. [27] performed joint optimization and obtained a great policy by proposing an algorithm that combines multiple agents with D3QN to maximize network utility and guarantee the quality of service (QoS).

Various schemes have been studied in relation to the problem of resource scheduling between different services. An innovative overlay/perforation framework [28,29] is based on the principle of overlaying a part of eMBB services when sporadic uRLLC services occur, although this approach may lead to significant degradation of the QoE of eMBB. Feng et al. [30] and Han et al. [31] designed a long and short dual time-scale algorithm for bandwidth allocation and service control, respectively, using Lyapunov optimization to centrally guarantee the latency of URLLC service while improving the quality of the eMBB continuous service. Han et al. [32] presented a dynamic framework of Q-learning based to improve the latency QoS and energy consumption ratio between URLLC and eMBB traffic in 5G/B5G. Moreover, Wang et al. [33] proposed a deep deterministic policy gradient (DDPG) algorithm to optimize the hole punch location and bandwidth allocation of URLLC services, and realize the QoS trade-off between URLLC and eMBB in 5G/B5G. Alsenwi et al. [34] used the DRL-based optimization auxiliary framework to solve the resource slicing problem in the dynamic reuse scenario of eMBB and URLLC services, achieved the ideal data rate of eMBB under the reliability constraints of URLLC and reduced the impact of URLLC traffic that was immediately scheduled on the reliability of eMBB. The time slots occupied with eMBB are split into the small slot and URLLC traffic pre-overlap at the small slot so that the proportional fairness of eMBB users can be maximized while satisfying the URLLC constraint [35]. Almekhlafi et al. [36] introduced a new technology that can reuse URLLC and eMBB services to reduce the size of perforated eMBB symbols, and improved the reliability of eMBB, symbol error rate (SER) and SE in the study to meet the delay constraints and reliability of URLLC. In [37], a new hybrid punching and coverage strategy is used to enhance the compromise between the acceptable number of URLLC users and the throughput of eMBB users.

As described in the abovementioned literature, RL is used to solve the dynamic resource allocation problem in various scenarios and has shown good performance. However, the performance requirements of URLLC are not prioritized and the resource scheduling problem among different services is not addressed. In addition, the traditional optimization algorithm and RL algorithm can be used to solve the resource scheduling problem between eMBB and URLLC services, but they still face a series of difficulties and challenges. For example, when scheduling resources among diverse services, the overlay/perforation framework has a huge influence on the QoS of eMBB in order to enhance the performance requirements of URLLC, and the Lyapunov dual time-scale algorithm improves the continuous QoS of eMBB, but its scheduling time slot is long and the optimization speed is slow. In this paper, a new Dueling DQN-based resource allocation and scheduling algorithm that satisfies the slice requirements is proposed. For the different demand characteristics of eMBB and URLLC services, especially for the ultra-low latency constraint of URLLC services, part of the bandwidth resources occupied by users of eMBB services are scheduled to URLLC users. With spectrum efficiency (SE) and quality of experience (QoE) of the two services as the optimization objectives, we have formed an optimization problem restricted by the rate and delay constraints of the two services and innovatively used Dueling DQN to solve the non-convex optimization problem of slicing resource allocation. Meanwhile, we use the resource scheduling mechanism and ϵ-greedy strategy to select the optimal resource allocation action, adopt the reward-clipping mechanism to enhance the optimization goal and select a reasonable bandwidth resolution (*b*) to improve the flexibility of bandwidth resource allocation. The main work can be summarized in three aspects.

(1) First, a scenario in which multiple wireless access network slices exist and BSs share bandwidth resources is considered. In this scenario, the resources are allocated and scheduled by BS for users with two different services. For the different demand characteristics of eMBB and URLLC services, especially for the ultra-low latency constraint of URLLC services, some of the bandwidth resources occupied by users of eMBB services are scheduled to URLLC users.

(2) Second, a novelty Dueling DQN-based algorithm aimed at allocating and scheduling of bandwidth resources is proposed. The problem regarding resource allocation and scheduling for eMBB and URLLC is modeled as an optimization problem and plotted as a Markov process, which is addressed through Dueling DQN training. Dueling DQN divides the action–value function output from the neural network into a state–value function and a dominance function, which reduces the correlation between the current state-action and action selection, and this network architecture is suitable for solving the proposed slicing resource allocation problem in discrete action space. More importantly, we generate the state using the number of packets received by two different service users and define the size of the bandwidth resources allocated to the two slices as actions. Since both the system SE and the QoE of eMBB and URLLC are optimization objectives, it is necessary to consider both the SE and the QoE. Therefore, the reward-clipping mechanism is proposed to encourage the agent to choose the best resource allocation action. Meanwhile, we choose the appropriate bandwidth allocation resolution to ensure the appropriate action space size and increase the flexibility of resource allocation.

(3) Third, the simulations are performed and reasonable data are obtained. It can be seen from the obtained data that the proposed algorithm ensures that the QoEs of eMBB and URLLC are stable at 1.0 with a high probability, meeting the service requirements in this scenario. Moreover, the QoE, SE and network utility show convergence trends. In contrast with Q-learning, DQN as well as Double DQN, the proposed Dueling DQN algorithm improves the network utility by 11%, 8% and 2%, respectively. Furthermore, the resource scheduling mechanism improves the QoE of URLLC, SE and network utility by 3%, 4.5% and 7%, respectively.

The organization of the following section in this paper is as follows. Section 2 builds the system model and formulates the optimization problem. In Section 3, the theoretical basis of Dueling DQN is introduced and the proposed slice resource allocation algorithm based on Dueling DQN is discussed in detail. Section 4 displays the simulation parameters and results. Finally, Section 5 concludes this work.

## 2. Problem Statements

### 2.1. System Model

Under the 3GPP standard, the main application scenarios of 5G networks include eMBB, URLLC and mMTC services, but they are widely different. The eMBB is a continuous wide-area coverage and high-capacity hot spot scenario. Its high-capacity hot spots mainly target at local hot spots, providing users with a high-speed data transmission rate and meeting users’ high traffic requirements. The URLLC can support ultra-high reliability connection under high-speed mobile conditions with extremely low latency. The mMTC has a large number of connected devices, but it typically sends relatively low amounts of non-latency-sensitive data. Obviously, eMBB and URLLC have higher bandwidth requirements and greater fluctuation in their resource requirements, while mMTC has fewer resource requirements and little fluctuation. Therefore, we do not consider the mMTC service of 5G network applications. This paper focuses on the problem of allocating limited bandwidth resources to URLLC and eMBB hybrid services and scheduling resources between slices. When the bandwidth resources provided by the BS to users are insufficient, the packet dropping probability increases for both eMBB and URLLC services, leading to a decrease in QoE. In order to improve the QoE of URLLC, SE and network utility on basis of ensuring the QoE of eMBB, the bandwidth allocation resolution (*b*) is chosen for the resource allocation and scheduling.

**Figure 1:**
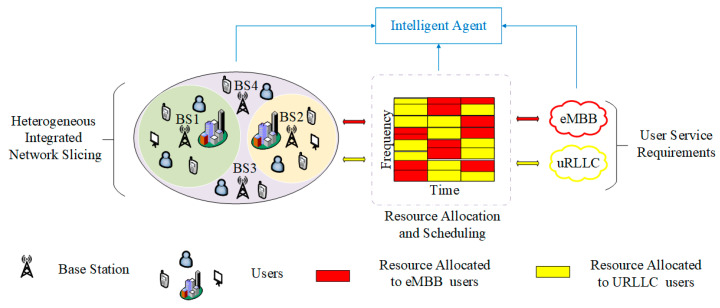
Network slicing scenario with multiple BSs and eMBB and URLLC users.

As presented in Figure 1, we take a scenario of heterogeneous integrated network slicing into account, which is composed of multiple BSs and users. The physical network is divided into N slices {1, 2,…,N}. The user set U contains M users $\left\{u_{1},u_{2}\;,\ldots ,u_{M}\right\}$, including J eMBB users and K URLLC users. In Figure 1, the network entity represented by the agent in the actual 5G network environment is the software-defined network (SDN), which obtains the information of the BS and users in the environment, controls the BS to slice and allocates bandwidth resources to users as required. The relationship between the agent and the whole environment is as follows. The agent can obtain the changes of eMBB and URLLC requirements and the resource allocation information of the BS in the environment in a timely manner. In the current time slot, when users of different services request resources from the BS, the agent uses the number of packets received by users of two different services to generate a state and defines the size of bandwidth resources allocated to the two slices as actions. Then, they traverse all actions in each state and select the best action according to the ϵ-greedy strategy, which corresponds to the bandwidth allocation scheme of BS. Meanwhile, agents form rewards based on the reward-clipping mechanism and obtain new states according to environmental changes.

#### 2.1.1. Resource Allocation of eMBB and URLLC Slices

In a heterogeneous integrated network slicing scenario with hybrid eMBB and URLLC services, both slices share all bandwidth resources. In order to denote the allocation of bandwidth resources, the binary variable $\lambda_{e\;}\in \left(0,1\right)$ and $\lambda_{l\;}\in \left(0,1\right)$ are defined, where $\lambda_{e\;}=1$ and $\lambda_{l\;}=1$ indicate that the bandwidth resources are allocated to users of eMBB and URLLC, respectively. The bandwidth allocated by the BS to eMBB users can be denoted by

(1) $$B_{u_{e}}=\lambda_{e\;}\cdot b\cdot d_{u_{e}}$$

where b denotes the bandwidth allocation resolution and $d_{u_{e}}$ indicates the amount of bandwidth allocated from the BS to eMBB users. The bandwidth obtained by the URLLC user from the BS is represented as

(2) $$B_{u_{l}}=\lambda_{l\;}\cdot b\cdot d_{u_{l}}$$

where $d_{u_{l}}$ is the amount of bandwidth allocated from the BS to URLLC users.

#### 2.1.2. Resource Scheduling between URLLC and eMBB Slices

When URLLC users request bandwidth resources from the BS and the bandwidth resources in the BS are already fully occupied by the eMBB slice and other URLLC users, part of the bandwidth resources occupied by the eMBB users will be scheduled by the BS to the URLLC users. The purpose of resource scheduling is to guarantee the QoE of URLLC. Define the total bandwidth requested by L(L≤K) URLLC users as $B_{e,l}$. To prevent the termination of partial eMBB service and reduce the impact of bandwidth resource scheduling on the QoE of eMBB due to bandwidth resource scheduling, the bandwidth occupied by any eMBB user will not be fully scheduled for the URLLC users. The bandwidth resources being scheduled for each eMBB user can be given as

(3) $$B_{u_{e},u_{l}}=b\cdot d_{e,l}$$

where $d_{e,l}$ denotes the number of bandwidth resources lost for each eMBB user. However, the resource scheduling must satisfy the following condition

(4) $$B_{u_{e}}-B_{u_{e},u_{l}}\geq B_{0}$$

where $B_{0}$ denotes the minimum bandwidth required to guarantee the minimum transmission rate for eMBB service. If L eMBB users are unable to provide bandwidth resources for the URLLC users, other L eMBB users will continue to provide bandwidth resources for the URLLC users.

The bandwidth resources obtained from the BS for eMBB and URLLC slices can be denoted as

(5) $$B_{e}=\sum_{u_{e}\in U}B_{u_{e}}-B_{e,l}$$

(6) $$B_{l}=\sum_{u_{l}\in U}B_{u_{l}}+B_{e,l}$$

In summary, the situation of the bandwidth resources allocated by the BS to the users is completed.

### 2.2. Problem Formulation

#### 2.2.1. Calculation of the SE

In the system with hybrid eMBB and URLLC services, improving resource utilization becomes a problem to be solved. Let SE denotes a symbolic evaluation index.

The noise in the Rayleigh fading channel cannot be ignored when the base station and the user are connected. However, in our environment, co-channel interference is avoided because each base station operates on a different frequency band to allocate bandwidth to users with different service types. Therefore, we use signal-to-noise ratio (SNR) when calculating the transmission rate. For the eMBB user, the transmission rate is related to the allocated bandwidth resources and the SNR between the BS and the user. It is calculated by

(7) $$r_{u_{e}}=B_{e,o}\log_{2}\frac{h_{e}\;\cdot \;p_{e}}{B_{e,o}\;\cdot \;N_{0}}\;$$

where $h_{e}$ denotes the channel gain between the BS and the eMBB user, $p_{e}$ represents the transmitted power of the BS connected to the eMBB user and $N_{0}$ is the noise power spectral density. However, $B_{e,o}$ has two cases, as follows. The bandwidth resources of eMBB users who lose resources are $B_{u_{e}}-B_{u_{e},u_{l}}$, and the bandwidth resources of the other eMBB users remain $B_{u_{e}}$. The sum of downlink transmission rate for eMBB users can be calculated as

(8) $$R_{e}=\sum_{u_{e}\in U}r_{u_{e}}$$

Similarly, we have the downlink transmission rate of URLLC user

(9) $$r_{u_{l}}=B_{l,o}\log_{2}\frac{h_{l}\;\cdot \;p_{l}}{B_{l,o}\;\cdot \;N_{0}}\;$$

where $h_{l}$ denotes the channel gain between the BS and the URLLC user and $p_{l}$ represents the transmitted power of the BS connected to the URLLC user. Moreover, $B_{l,o}$ also has two cases, as follows. For URLLC users who obtain resources through scheduling, their bandwidth resources are $B_{u_{l}}+B_{u_{e},u_{l}}$, while other URLLC users maintain their bandwidth resources at $B_{u_{l}}$. We can calculate the sum of the downlink transmission rate for URLLC users as

(10) $$R_{l}=\sum_{u_{l}\in U}r_{u_{l}}$$

since the SE is considered the sum of the downlink transmission rate divided by the total bandwidth (*W*) allocated from the BS to the users. The SE can be denoted by a variable Y and formulated as

(11) $$Y=\frac{R_{e}+R_{l}}{W}\;$$

#### 2.2.2. Calculation of the QoE

Due to its requirements for low latency and ultra-reliability, we will prioritize the reduction of packet dropping probability for URLLC services. When the bandwidth resources in the current time slot are insufficient, the BS will partially schedule the bandwidth resources occupied by the transmission of eMBB packets to the URLLC users until the resources required for the transmission of URLLC packets are satisfied. The major objective of both services is to obtain low packet dropping probability and high transmission rates. Before quantifying the QoE, we define a binary variable ρ∈(0,1). When the packet transmission is successful, ρ is taken as 1, and when the packet transmission fails, ρ is taken as 0.

Let the number of packets transmitted (pkt) of eMBB users expressed as $p_{u_{e}}$. So, the total number of packets transmitted of eMBB users can be calculated as

(12) $$p_{e}=\sum_{u_{e}\in U}p_{u_{e}}$$

As the QoE is defined as the packet dropping probability, we define $q_{u_{e}}$ as a packet transmitted by an eMBB user. We can formulate the QoE of eMBB as

(13) $$Q_{e}=\frac{\sum_{u_{e}\in U}\sum_{q_{u_{e}}\in p_{u_{e}}}\rho }{p_{e}}\;$$

Moreover, we denote the pkt of URLLC users as $p_{u_{l}}$, and calculate the total number of packets transmitted of URLLC users by

(14) $$p_{l}=\sum_{u_{l}\in U}p_{u_{l}}$$

The $q_{u_{e}}$ is defined as a packet transmitted by an URLLC user. The QoE of URLLC is formulated as follows

(15) $$Q_{l}=\frac{\sum_{u_{l}\in U}\sum_{q_{u_{l}}\in p_{u_{l}}}\rho }{p_{l}}$$

#### 2.2.3. Calculation of the Network Utility

To address the resource allocation and scheduling problem in a hybrid services system, we achieve a reasonable allocation of resources in a diverse services system by dynamically adjusting the allocation of bandwidth resources for each slice. The optimization objective is the weighted sum of the SE and QoE of the two services, which we define as the network utility function F.

Mathematically, the formulated problem concerning the allocation and scheduling of resources is presented by

(16) $$\max_{B_{u_{e}},B_{u_{l}}}F=\alpha Y+\beta Q_{e}+\eta Q_{l}$$

(16a) $$S.\;t.\;\;\;B_{e}+B_{l}\leq W$$

(16b) $$B_{u_{e}}\leq C_{e}$$

(16c) $$B_{u_{l}}\leq C_{l}$$

(16d) $$r_{u_{e}}\geq \bar{r_{u_{e}}}$$

(16e) $$r_{u_{l}}\geq \bar{r_{u_{l}}}$$

(16f) $$t_{u_{e}}\leq \bar{t_{u_{e}}}$$

(16g) $$t_{u_{l}}\leq \bar{t_{u_{l}}}$$

where α, β,η denote the importance weight values of the SE, the QoE of eMBB $Q_{e}$ and the QoE of URLLC $Q_{l}$, respectively. The total number of bandwidth resources obtained from the BS for both eMBB and URLLC slices less than the total bandwidth resources of the system is denoted in Equation (16a). Equations (16b) and (16c) indicate that the bandwidth resources allocated by the BS for both eMBB and URLLC slices must not exceed the eMBB and URLLC downlink bandwidth capacity $C_{e}$ and $C_{l}$, respectively. The transmission rate of eMBB and URLLC users must be higher than the transmission rate specifications of both services in 5G scenarios $\bar{r_{u_{e}}}$ and $\bar{r_{u_{l}}}$, which are expressed in Equations (16d) and (16e). Additionally, Equations (16f) and (16g) denote that the eMBB and URLLC services transmission latency must be lower than the maximum latency requirement $\bar{t_{u_{e}}}$ and $\bar{t_{u_{l}}}$ in 5G scenario, respectively.

In the heterogeneously integrated network slicing scenario in which eMBB and URLLC service requirements coexist, the resource scheduling and allocation processes of front and rear timeslots interact. The resource allocation in each time slot should meet the current fluctuating demand. However, with the time-varying nature of both the remaining resources and user demand, the BS needs to continuously change the resource allocation scheme to ensure the QoE for both services and improve SE and network utility. To illustrate that the optimization problem formed is non-convex and NP-Hard, it is mapped to the 0–1 backpack problem. Assume a backpack of capacity $C_{n}$ and $T_{n}$ items and define the value of each item to be $p_{n}$ and the weight to be $w_{n}$. The purpose is to search for a subset $T_{n}^{\prime }\in T_{n}$ that obtains the maximum $\sum_{T_{n}^{\prime }\in T_{n}}p_{n}$ under the condition that $\sum_{T_{n}^{\prime }\in T_{n}}w_{n}\leq C_{n}$. Meanwhile, a simplified form of the optimization problem in this paper is considered, i.e., the case in which only one slice of URLLC is available. Then, the optimization objective of this simplified problem can be denoted as

(17) $$\max_{B_{u_{l}}}F_{l}=\alpha Y+\eta Q_{l}=\alpha \frac{R_{l}}{B_{l}}+\eta \frac{\sum_{u_{l}\in U}\sum_{q_{u_{l}}\in p_{u_{l}}}\rho }{p_{l}}$$

(17a) $$\;S.\;t.\;B_{l}\leq W$$

(17b) $$B_{u_{l}}\leq C_{l}$$

(17c) $$r_{u_{l}}\geq \bar{r_{u_{l}}}$$

(17d) $$t_{u_{l}}\leq \bar{t_{u_{l}}}$$

Mapping the 0–1 backpack problem to this optimization objective, the quantity of items $T_{n}$ corresponds to the users in URLLC slice, the value $p_{n}$ corresponds to the weighted sum of QoE and SE achieved by the slice and the weight $w_{n}$ corresponds to the limit of downlink capacity, transmission rate, and latency. Obviously, the optimization problem can be completed in one polynomial time, while the 0–1 backpack problem has NP-Hard characteristics, so the simplified problem can be classified as an NP-Hard problem. It can be concluded that the optimization problem formed in this paper is a non-convex optimization and is NP Hard. Since the prior transfer probability is unknown, it is very difficult to obtain the closed optimal solution of the formulaic problem. However, RL is more suitable for solving such problems in which the probability of prior transfer is unknown. Therefore, we use RL to find the best scheme for the allocation and scheduling of bandwidth resources in heterogeneous integrated networks.

## 3. Proposed Algorithm

### 3.1. Foundation of Dueling DQN

It is worth mentioning that DQN, as a branch of DRL, uses two key technologies for improvement and has outstanding advantages in decision making. Firstly, the experience replay mechanism breaks the inherent correlation among samples, making them independent of each other. Secondly, the target value network can enhance the convergence and stability of training by lessening the correlation between the current and target Q value, correspondingly. An intelligent agent obtains information about the environment through trial and error and uses the data obtained during the interaction as observations. Then, the agent traverses the actions in a given state and finds the corresponding action with the largest Q value according to its ϵ-greedy policy. However, DQN has a disadvantage in that the Q value output by its neural network denotes the selected action value in the state, which is dependent on the action and state. This means that the DQN fails to reflect the different effects of state and action on the Q value. Furthermore, DQN is vulnerable to the overestimation problem, resulting in poor training stability. Among the improvements of DQN in recent years, Dueling DQN has outstanding advantages, and its network stability and convergence speed have been significantly improved. In particular, Dueling DQN maintains the advantages of the DQN while improving on the DQN in terms of network structure by dividing the action–value function from the output of the neural network into the state–value function and advantage function. This allows Dueling DQN to learn the value function for each state without considering what action to take in that state. Therefore, the Dueling DQN converges better when the current action is less relevant to the successive state and the current state–action function is also less relevant to the current action selection. Based on the improvements and advantages of Dueling DQN over DQN, we prefer to choose Dueling DQN for iterative optimization of proposed nonconvex optimization problems.

The process of interaction between the agent of Dueling DQN and the environment can be cast into a Markov decision process (S,A,R,P,γ), where S presents the state space, A is the action space. The current state s and the next state $s^{\prime }$ are stored in the state space, while the current action a and the next action $a^{\prime }$ are stored in the action space. R denotes the reward function, which is the goal that the agent maximizes during action selection and is the key factor that makes the training process more stable. P is the transfer probability, which represents the probability that the current state will be transferred to another state when an action is performed. γ is a discount factor greater than 0 and less than 1 that moderates near and far-term effects in reinforcement learning. The action–value function can be formulated as

(18) $$Q^{\pi }\left(s,a\right)=V^{\pi }\left(s\right)+A^{\pi }\left(s,a\right)$$

Here, the policy π denotes the distribution that maps state to action. Then, the two functions $A^{\pi }\left(s,a\right)$ and $V^{\pi }\left(s\right)$ are approximated using the neural network. It can be seen that $V^{\pi }\left(s\right)$ relates only to states, while $A^{\pi }\left(s,a\right)$ relates to both states and actions. In fact, there are two neural networks with parameters θ in the Dueling DQN: the target Q network and the evaluation Q network, respectively. Let Q(s,a;θ,ϕ,φ) denote the value function with parameters θ, which is expressed as

(19) Q(s,a;θ,ϕ,φ)=V(s;θ,ϕ)+A(s,a;θ,φ)

where θ is a shared parameter, ϕ denotes a dominant function parameter and φ is used to indicate a parameter of the action–value function. However, there exists a serious problem in the above equation, which is that the unique V(s;θ,ϕ) and A(s,a;θ,φ) cannot be obtained from Q(s,a;θ,ϕ,φ) in Equation (19). Thus, a centralization processing of the advantage function is performed to guarantee that zero dominance will occur for a given action. Further, Q(s,a;θ,ϕ,φ) can be reformulated as

(20) $$Q\left(s,a;\theta ,\phi ,\varphi \right)=V\left(s;\theta ,\phi \right)+\left[A\left(s,a;\theta ,\varphi \right)-\frac{1}{\left|A\right|}\sum_{a^{\prime }}A\left(s,a^{\prime };\theta ,\varphi \right)\right]$$

The agent of Dueling DQN makes an observation as it interacts with the environment. The Q-network calculates all Q values for each action when observation is used as state inputs. Then, the agent selects the action that maximizes the Q value relying on a ϵ-greedy strategy and provides the reward value. In Dueling DQN, the target Q value of the target Q-network is updated by copying the current Q value every C iterations. However, the current Q value is reset with real-time updates in each iteration. The target Q value ($Q_{t}$) of the target Q-network is denoted by

(21) $$Q_{t}=r+\gamma \max_{a\prime }\hat{Q}\left(s^{\prime },a^{\prime };\theta ,\phi ,\varphi \right)$$

Then, the loss function L(θ) in Dueling DQN is defined by

(22) $$L\left(\theta \right)=E\left[{\left(Q_{t}-Q\left(s,a;\theta ,\phi ,\varphi \right)\right)}^{2}\right]$$

where E denotes the expected value. Meanwhile, the optimal parameter θ is obtained through the minimization of the square of TD error; that is,

(23) $$\varsigma^{2}={\left[Q_{t}-Q\left(s,a;\theta ,\phi ,\varphi \right)\right]}^{2}$$

Finally, the action–value function Q(s,a;θ,ϕ,φ) is updated by

(24) $$Q\left(s,a;\theta ,\phi ,\varphi \right)=Q\left(s,a;\theta ,\phi ,\varphi \right)+\varepsilon \left[Q_{t}-Q\left(s,a;\theta ,\phi ,\varphi \right)\right]$$

The iterative training of Dueling DQN requires that a fixed number of iterations be set. When the iteration is ended, Dueling DQN can utilize the trained neural network for optimal action selection.

### 3.2. The Dueling DQN Based Slicing Resource Allocation and Scheduling Algorithm

The proposed Dueling DQN-based algorithm is used for resource allocation and scheduling in eMBB and URLLC hybrid traffic. Bandwidth resources are dynamically allocated and scheduled so that the requirements of users are better met and network utility is maximized. In cases in which the bandwidth resources are insufficient, the BS schedules some of the bandwidth resources occupied by eMBB service to the URLLC service, improving the QoE of URLLC and network utility with the premise of ensuring the QoE of the eMBB.

To achieve resource scheduling between eMBB and URLLC users, the following resource scheduling mechanism is set up. When randomly distributed users request resources from the BS, the BS counts the number of users requesting resources and slices the bandwidth by service types. In each iteration, the BS allocates resources to the users of both services while counting the number of users that request resources. When there are insufficient bandwidth resources and URLLC users still request resources, the BS schedules bandwidth resources occupied by the corresponding number of the users of eMBB to URLLC based on the number of URLLC users requesting resources. Furthermore, the bandwidth resources occupied by eMBB users are not all scheduled to avoid service interruption for this user. After several iterations, a resource scheduling scheme can be obtained to provide the best network utility.

The goal of this paper is to improve the SE of the network system while guaranteeing the QoE of both services, so we use a reward-clipping mechanism that allows the agent to optimize both metrics through the algorithm. Since the QoE and the SE of the system are of different orders of magnitude, we use different coefficients in each segment of the reward parameter to make the reward value reach a value that is easy for the agent to simulate and learn. We expect users of both services to achieve satisfactory QoE. In order to ensure that the QoE of slices meet the 5G standard and reach 1.0 as often as possible, we set the QoE threshold of 0.98 in the reward function. If the $Q_{l}$ cannot satisfy the requirement we mentioned above, a more unfavorable negative reward value in Equation (25) will be calculated by

(25) $$r=-3+\left[\left(Q_{l}-1\right)\;\cdot \;10\right]$$

When the $Q_{l}$ satisfies the requirement but the $Q_{e}$ cannot, we will have a negative reward value as follows

(26) $$r=\left(Q_{l}-1\right)\;\cdot \;10$$

Similarly, we would like to see an improvement in SE. We compared the highest value and lowest value of SE in the algorithm training process, and in order to ensure a higher system SE is achieved as often as possible, so that the agent presents a stable training trend in the training process, we set SE as 380 between the highest value and the lowest value in the reward function. If the QoE of both services can be achieved but the SE does not satisfy this condition, a poorer positive reward value can be given as

(27) r=Y · 0.01

Conversely, the QoE of both services can be achieved and the SE satisfies this condition; thus, we will calculate a better positive reward value as follows:

(28) r=5+[(Y−380) · 0.1]

**Figure 2:**
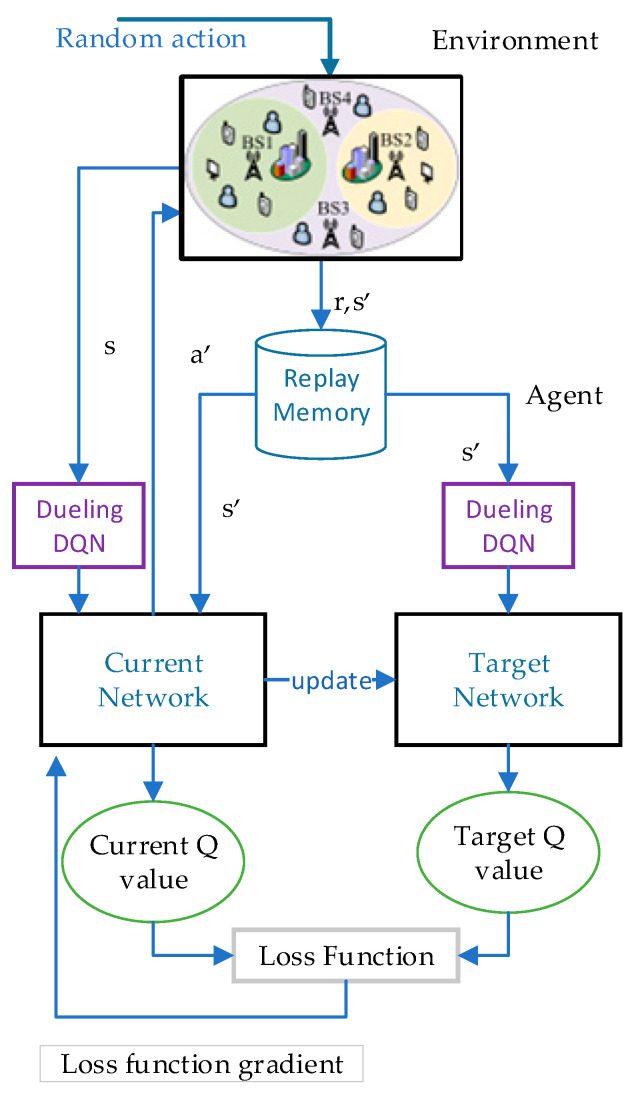
Algorithm flow diagram.

The procedures of resource allocation and scheduling for hybrid eMBB and URLLC services using the proposed Dueling DQN based algorithm with resource allocation and scheduling are as follows. In order to helps readers to understand our process more clearly, the algorithm flowchart is shown in Figure 2.

Before starting the iterative training, the parameters are initialized and a randomly selected policy is required to produce an original state. Moreover, the BS randomly selects an allocation scheme to first allocate bandwidth resources for eMBB and URLLC users, and then schedule the bandwidth resources according to the resource scheduling mechanism. After the end of scheduling, the intelligent agent of the Dueling DQN obtains information during its interaction with the environment and calculates the pkt of eMBB and URLLC users as an observation. Afterward, the observation is entered into the Q-network to form the initial state.

Each iteration performs the operations as follows. The BS selects a resource allocation action based on the policy in the Dueling DQN, after which scheduling is performed. Then, the user receives the resource allocated by the BS, and S and A are updated in the Dueling DQN. Each state in the state space is the number of eMBB and URLLC packets successfully transferred. Each action in the action space is the bandwidth resource allocated to users by BS based on the network utility and the state feedback from users. The SE and the packet dropping probabilities are calculated according to Equations (11), (13) and (15). Thereby, the network utility can be calculated as shown in Equation (16). It is worth mentioning that the reward calculation formula is one of Equations (25)–(28). Once again, the pkt is calculated as the next state. Then, the $s,a,s^{\prime },r$ is imported to Dueling DQN for training.

For each iteration, the training process is as follows. Firstly, the agent obtains $s,a,s^{\prime },r$ in the response of the environment, which is saved in the replay memory as a transfer sample. After enough data are deposited in the sample pool, a minibatch-sized transaction is randomly selected in the sample pool for training. Secondly, the evaluation Q-network of the agent adds the advantage function of centralized processing to the state-value function to obtain the current Q value, as illustrated in Equation (20). Meanwhile, Equation (21) is the formula used by the intelligent agent to calculate the target Q value. Moreover, the action that maximizes the current Q value in a given state is selected on the basis of the ϵ-greedy strategy. Finally, the current update of Q network parameters is based on the loss function in Equation (22) and the gradient descent method in Equation (23). Consistent with Equation (24), the current Q-network parameters are cloned into the target Q-network by resetting to complete the parameter update of the target Q-network after *C* iterations.

Using the predetermined number of iterations, the value function network with great performance is trained. The Dueling DQN is capable of obtaining an action under the ϵ-greedy strategy for a given state to reduce the loss function and improve the cumulative expected reward. Therefore, the best scheme of resource allocation and scheduling can be obtained in the eMBB and URLLC hybrid service system, which improves the QoE of URLLC, SE and network utility while ensuring the QoE of eMBB. The pseudocode of the proposed algorithm is presented in Algorithm 1.
**Algorithm 1.** The Dueling DQN based slicing resource allocation and scheduling1:**Initialize** the replay memory D, the capacity P, the current and target action-value function Q and $\hat{Q}$ with random weights θ, the parameter ϕ and φ;2:Choose random action $a_{0}$ to allocate bandwidth for eMBB and URLLC users;3:**Scheduling:**4:    User ← The bandwidth resources;5:  The URLLC users continue to request resources;6:  The eMBB users $\overset{b}{\to }$ The URLLC users;7:The pkt calculated →s;8:**Repeat**9:  **For** iteration = 1, to *T*, **do**10:    Policy → a choosed;11:    Execution **Scheduling**;12:    The SE is calculated as shown in Equation (11);13:    The $Q_{e}$ and $Q_{l}$ are calculated on the basis of Equations (13) and (15);14:    Calculate the network utility based on Equation (16); 15:    Calculate the reward based on one of Equations (25)–(28);16:    The pkt calculated $\to s^{\prime }$;17:      **#** Train Dueling DQN;18:      The $\left\{s,a,s^{\prime },r\right\}$ is stored in D of Dueling DQN;19:The agent samples $\left\{s_{i},a_{i},s_{i+1},r_{i}\right\}\;$ from D;20:      Define $Q\left(s_{i},a_{i};\theta ,\phi ,\varphi \right)$ according to Equation (20); 21:      Set $$y_{i}=\{\begin{array}{c} r_{i}\\ r_{i}+\gamma max_{a^{\prime }}\hat{Q}\left(s_{i+1},a^{\prime };\theta^{*},\phi ,\varphi \right) \end{array}\begin{array}{c} \mathrm{if}\;\mathrm{terminal}\;\mathrm{step}\;i+1\\ \mathrm{otherwise} \end{array}$$
22:      The agent updates the network parameters θ by ${\left[y_{i}-\hat{Q}\left(s_{i},a_{i};\theta ,\phi ,\varphi \right)\right]}^{2}$;23:      Executed $\hat{Q}\leftarrow Q$ every *C* iterations;24:  
**End for**
25:**Until** The end of the iterations.

### 3.3. Time Complexity Analysis of Algorithm

The time complexity of the training phase needs to consider the time complexity of training the Q network and the number of attempts needed to train the Q network. In the process of training the Q network, the connection weights between every two adjacent layers of neurons need to be updated. We set the number of layers of the Q network as $x_{i}$, the number of neurons in the ith layer to be $n_{i}$, and the number of iterations in each training to be $t_{train}$, then the time complexity $c_{train}$ of training a Q network once can be calculated as.

(29) $$c_{train}=t_{train}D\left(\overset{x=1}{\underset{i=1}{\sum }}x_{i}x_{i+1}\right)$$

We denote the total number of iterations in the algorithm as $t_{total}$, and the number of steps in each iteration as $t_{step}$, then the number of times to train the Q network is $t_{total}\cdot t_{step}$, so the time complexity of the proposed algorithm training phase can be calculated as

(30) $$c_{train}=t_{total}\cdot t_{step}\cdot t_{train}D\left(\overset{x=1}{\underset{i=1}{\sum }}x_{i}x_{i+1}\right)$$

The time complexity of the online training phase of the deep reinforcement learning algorithm is high, but after the Q network is trained, the Q network does not need to be updated in the running phase, and the time complexity is low, which can meet the requirements of online decision-making time under real-time network conditions. Since the algorithms we compared in the simulation are all deep reinforcement learning algorithms and set the same parameters, they are roughly the same in terms of algorithm complexity.

## 4. Simulation Results and Analysis

### 4.1. Simulation Parameters

In this part, we conduct extensive simulations to verify the performance of the algorithm we proposed in heterogeneous integrated networks. The simulation runs on a PC with an Intel Core i7-10750 H CPU at 2.6 GHz. The graphics card we use is NVIDIA GeForce GTX1650 Ti with 4G memory. We use TensorFlow 1.15 deep learning framework and Python 3.8 to implement our algorithm in the simulation. The scenario contains two kinds of services and two types of slices, correspondingly. In order to fit the actual situation and reflect the advantages of the algorithm, we set the available bandwidth *W* of BS to 20 MHz, the radius of BS to 50 m, and the number of users as 500. We set the rate and delay thresholds of eMBB and URLLC according to the 5G service level agreement. In order to ensure that the action space is not too large and better actions can be obtained, the bandwidth resolution is set to 0.1 MHz. In the actual scenario, the number of users of the eMBB service is often greater than the number of URLLC services, so the proportion of the number of eMBB and URLLC users is set to 3:2. For Rayleigh fading, we set the noise power spectral density to −174 dBm/Hz. The details of distribution of users and *pkt* standards according to [22] are listed in Table 1.

### 4.2. Performance Evaluation

Next, we illustrate the performance simulation results of the proposed Dueling DQN-based algorithm in detail. Furthermore, it is compared with different environmental parameters and with algorithms based on Q-learning, DQN as well as Double DQN. The Ref. [12] applies Q-learning to resource allocation in a network slicing scenario. In Refs. [21,22], the resource allocation algorithm based on DQN is mentioned and used as a comparison of resource allocation schemes in network slicing scenario. The algorithm based on Double DQN is used to solve the management and allocation of wireless network resources in Refs. [23,25]. In particular, the same learning ratios are set for Q-learning, DQN, Double DQN and Dueling DQN. For the common parameters of the algorithm, we set the learning rate to 0.01, the discount factor to 0.9, and the minimum batch size to 32 after our experiment. To unify the SE and QoE orders of magnitude and thus obtain easily comparable system utilities, the weights of the optimization target SE and QoE of both services are set as α = 0.01, β = 1, η = 3, respectively. The performance simulation results and brief analysis in terms of the QoE, SE and network utility are as follows.

**Figure 3:**
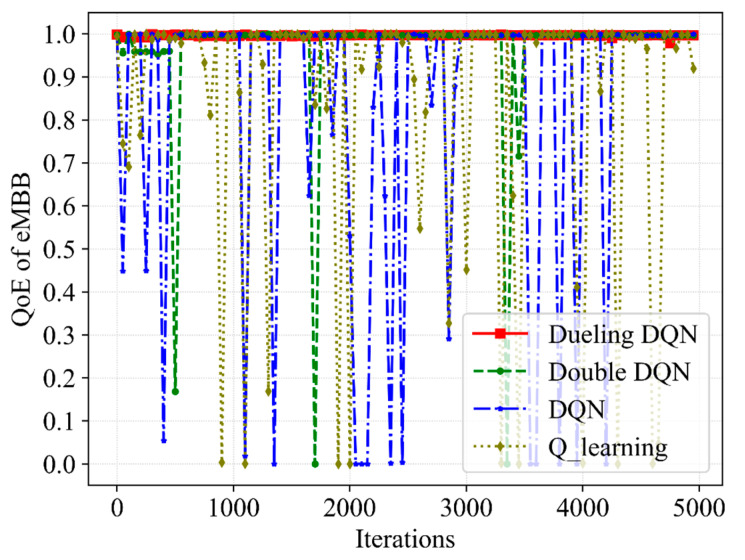
A comparative result of QoE for eMBB service with different slicing algorithms.

**Figure 4:**
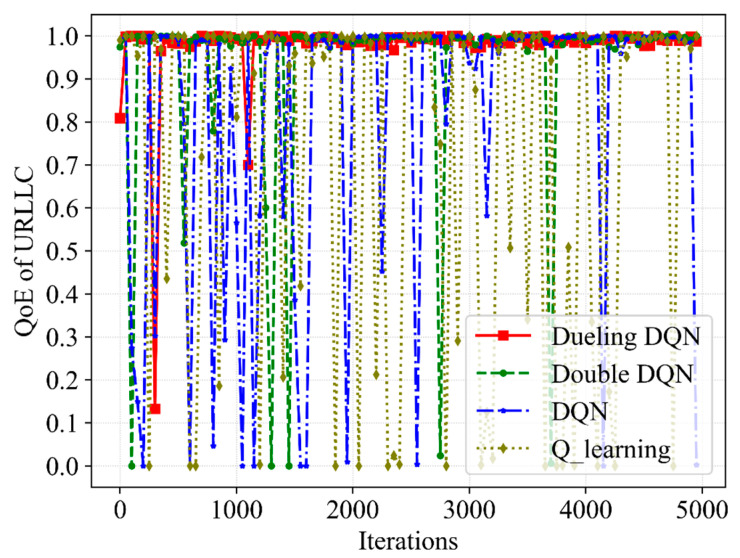
A comparative result of QoE for URLLC service with different slicing algorithms.

**Figure 5:**
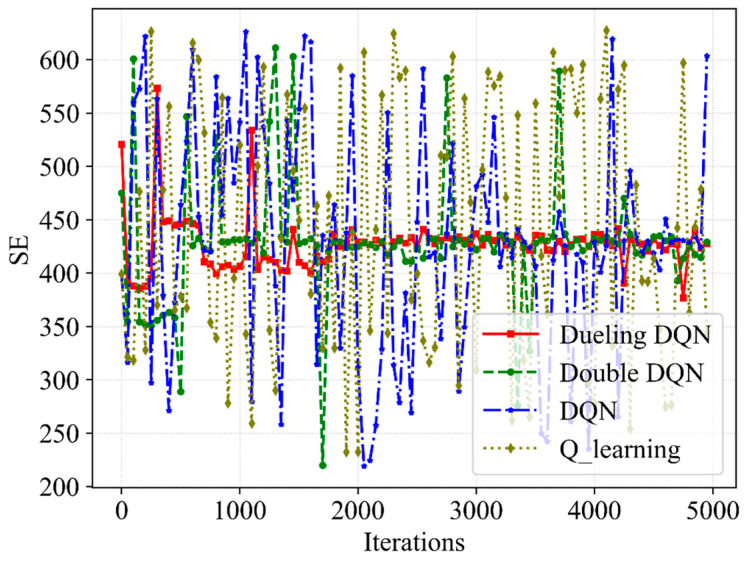
A comparative result of SE with different slicing algorithms.

**Figure 6:**
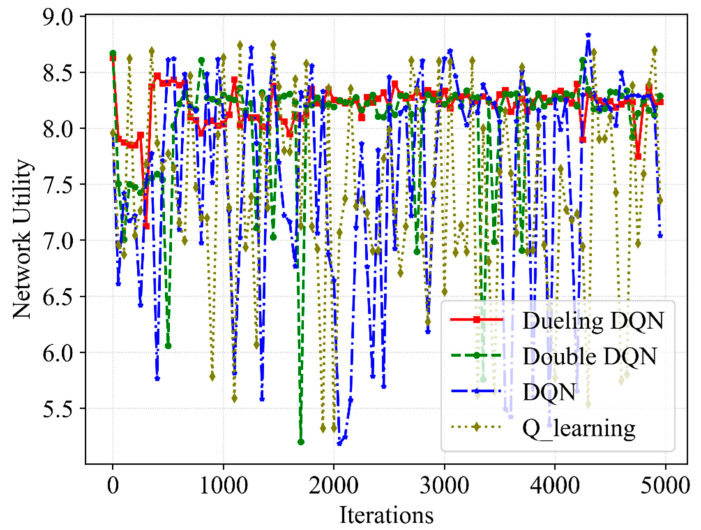
A comparative result of network utility with different slicing algorithms.

Figure 3, Figure 4, Figure 5 and Figure 6 present the performance of the QoE, SE and network utility with the slicing resource allocation algorithms based on Q-learning, DQN, Double DQN and Dueling DQN, respectively. Figure 3 and Figure 4 depict the tendency of QoE for both services with the increasing number of iterations. After about 1000 iterations, although the latency requirement of URLLC is higher than that of eMBB, the Dueling DQN can achieve almost 100% QoE of both services. Compared with Dueling DQN, Double DQN shows slightly less stability for both services, while both DQN and Q-learning show extreme instability for both services. The reason for this is that the Dueling DQN makes improvements compared with the other three algorithms, thereby allocating bandwidth resources more rationally and achieving the desired QoE.

Figure 5 presents the trend of SE with the increasing number of iterations. It is indicated that the SE obtained through Dueling DQN can be divided into three stages. The SE is high but unstable in the first 600 iterations, low from 600 iterations to 2000 iterations and basically stable at 430 after around 2000 iterations. This is due to the fact that the number of eMBB users is larger than the number of URLLC users and the BS allocates more bandwidth resources to eMBB users at the beginning of the iteration. In addition, the transmission rate achieved by eMBB users with the same bandwidth is much higher than the transmission rate achieved by URLLC users. Therefore, a large SE is calculated according to Equation (15). Since the proposed algorithm focuses on improving the QoE of URLLC, BS allocates more bandwidth resources to URLLC users after 600 iterations, reducing SE to about 400. After around 2000 iterations, the neural network parameters of the Dueling DQN are updated to a basically stable state, thereby obtaining the high and convergent SE. During the optimization process, SE has some values exceeding 450, which obtain very low network utility and reward because SE cannot guarantee the corresponding service quality. Therefore, the proposed algorithm does not allow the SE to converge to the anomaly height value described above. Through these too high and low abnormal values, we can see the advantages of the proposed Dueling DQN-based algorithm, which improves the SE while prioritizing the service quality. However, the unusual and sudden drop in performance late in the training period is due to the tiny non-zero ϵ-greedy exploration rate. Moreover, it is also possible to obtain the information that the Double DQN obtains the optimal curve of SE close to Dueling DQN but with greater fluctuation, while the other two algorithms have no convergence tendency. The Dueling DQN achieves the best convergence SE among the four algorithms, indicating that Dueling DQN can achieve the purpose of guaranteeing QoE and improving SE for different services by flexibly adjusting the resource allocation and scheduling.

Figure 6 further presents the tendency of the network utility as the increasing number of iterations. We define network utility as the weighted sum of SE and QoE, and QoE converges to 1.0 with high probability. Thus, the network utility has a strong correlation with the optimization of SE, which can also be divided into three stages. Under the influence of unstable SE and QoE, the network utility fluctuates widely at the beginning of the iteration. Although 100% QoE is achieved, the network utility shows a downward trend due to resource scheduling. Under the effect of reward, QoE and SE are improved and remain convergent, so the network utility can basically remain above 8.3 after 2000 iterations. The abnormally high SE in Figure 5 corresponds to the low network utility in Figure 6, which further explains that just a high SE cannot guarantee the service quality of eMBB and URLLC users. However, the Double DQN eventually converges to a value similar to the Dueling DQN but shows less stability, and both Q-learning and DQN show no apparent signs of convergence. Furthermore, in contrast with Q-learning, DQN as well as Double DQN, the proposed algorithm enhances the network utility by 11%, 8% and 2%, respectively.

**Figure 7:**
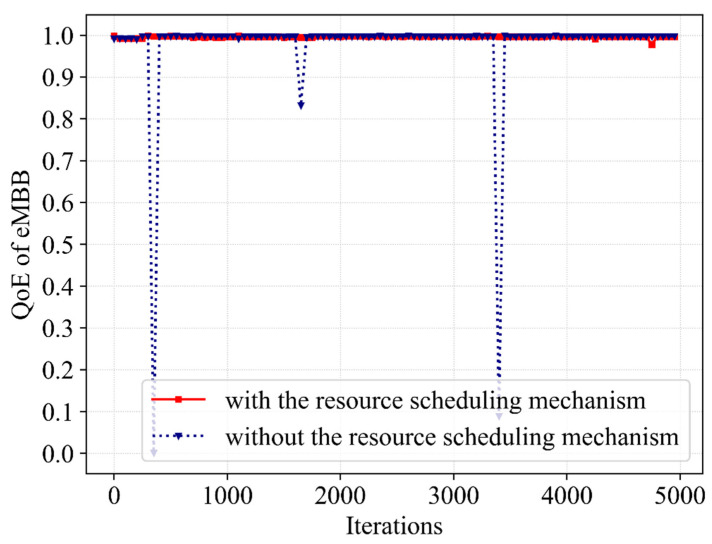
A comparative result of QoE for eMBB service with and without the resource scheduling mechanism.

**Figure 8:**
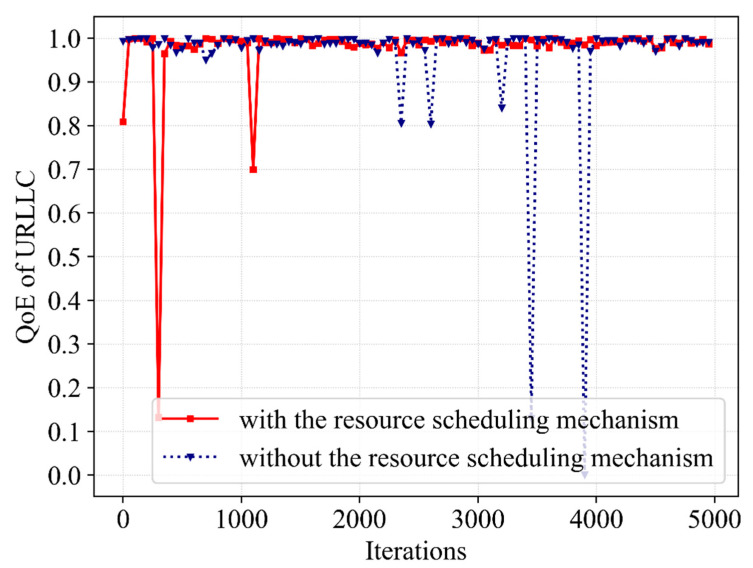
A comparative result of QoE for URLLC service with and without the resource scheduling mechanism.

Afterward, the impact of resource scheduling mechanisms on performance is further investigated. Figure 7 and Figure 8 show the differences in QoE of eMBB and URLLC during the iterative learning of Dueling DQN with and without the resource scheduling mechanism. For the eMBB users, when there is a resource scheduling mechanism, the QoE demonstrates trivial improvement. With the bandwidth resources which are allocated to eMBB users are much greater, the QoE of URLLC occasionally has values that fail to satisfy service requirements in the absence of the mechanism. Moreover, the QoE of URLLC is raised by 3% with the mechanism and there are no low outliers that do not satisfy URLLC feature requirements after optimization. This phenomenon occurs because some of the bandwidth resources occupied by eMBB are scheduled to URLLC in order to increase the bandwidth resources available for the transmission of URLLC packets without interrupting eMBB user services. Therefore, it ensures a low packet dropping probability of eMBB and reduces the packet dropping probability of URLLC. In other words, it improves the convergence stability of the QoE of the URLLC without reducing the QoE of the eMBB.

**Figure 9:**
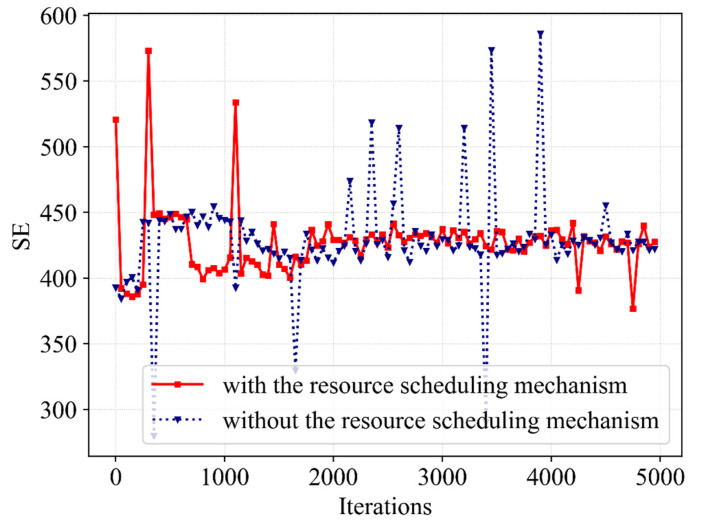
A comparative result of SE with and without the resource scheduling mechanism.

Figure 9 presents the impact on SE with and without the resource scheduling mechanism. The SE curve without a resource scheduling mechanism shows a trend of increasing first and then decreasing. At the beginning of iterative learning, the agent explores actions beneficial to SE, making SE improve to a better value. However, after iterative learning, it drops and remains at a low value, and occasionally shows a high outlier. When this mechanism is not considered, BS tends to allocate bandwidth in proportion to users, and the demand rate of eMB service is much higher than that of the URLLC service, resulting in high SE. Then, it learns to allocate bandwidth in a more rational manner after iterative training; that is, the bandwidth allocated to URLLC increases. The above factors cause the SE to drop slightly, and thus it fails to converge at higher values. Additionally, we can see that since the bandwidth resources allocated to eMBB are increased at this time, excessive SE values are obtained late in the iteration, corresponding to the low abnormal QoE of URLLC. However, the SE with the mechanism only obtains an excessive SE value at the beginning of the iteration because the QoE of URLLC is not satisfied, and tends to be stable after iterative learning. Meanwhile, the algorithm with the resource scheduling mechanism has a slightly faster convergence speed and better stability and can converge to a better value without outliers. Therefore, we can conclude that through the resource scheduling mechanism, the Dueling DQN can learn more rational bandwidth allocation to improve the stability of SE under the premise of guaranteeing the QoE of different services.

**Figure 10:**
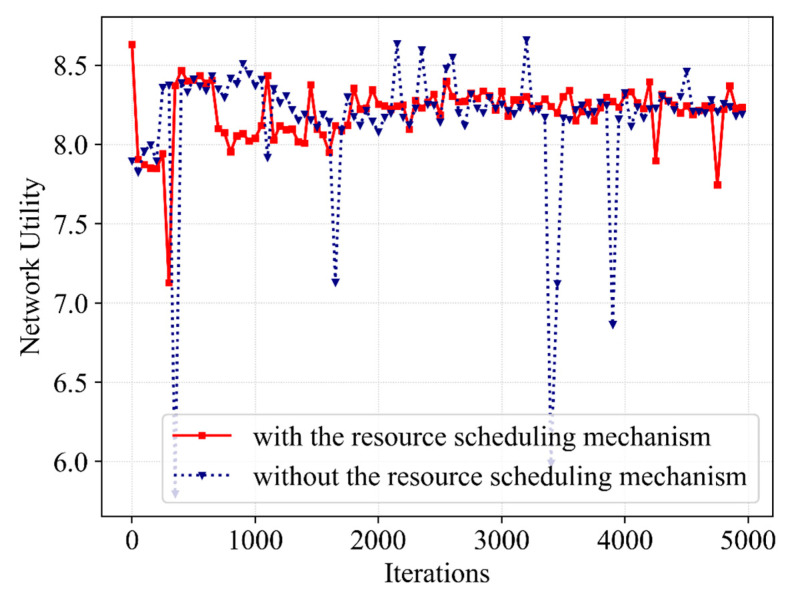
A comparative result of network utility with and without the resource scheduling mechanism.

Furthermore, Figure 10 continues to present the differences in network utility with and without the resource scheduling mechanism. Since QoE achieves the optimal value of 1 with a high probability, there is a strong correlation between network utility and SE. The network utility without a resource scheduling mechanism showed a high value in the first 2000 iterations but decreased slightly and remained at a low value in the subsequent iterations. During the iteration, the outlier in network utility is due to the high SE obtained by allocating more bandwidth resources to eMBB users, but at this time, the QoE of URLLC is not satisfied and the reward-clipping mechanism presents a worse reward value. However, the next bandwidth allocation action requires a great reward; thus, the resource allocation action is improved to satisfy the QoE of the URLLC and restore stability to the SE and network utility. Figure 7, Figure 8, Figure 9 and Figure 10 show that the utilization of the resource scheduling mechanism improves the SE and network utility by 4.5% and 7%, respectively. The simulation curves prove the effect of Dueling DQN with the resource scheduling mechanism.

**Figure 11:**
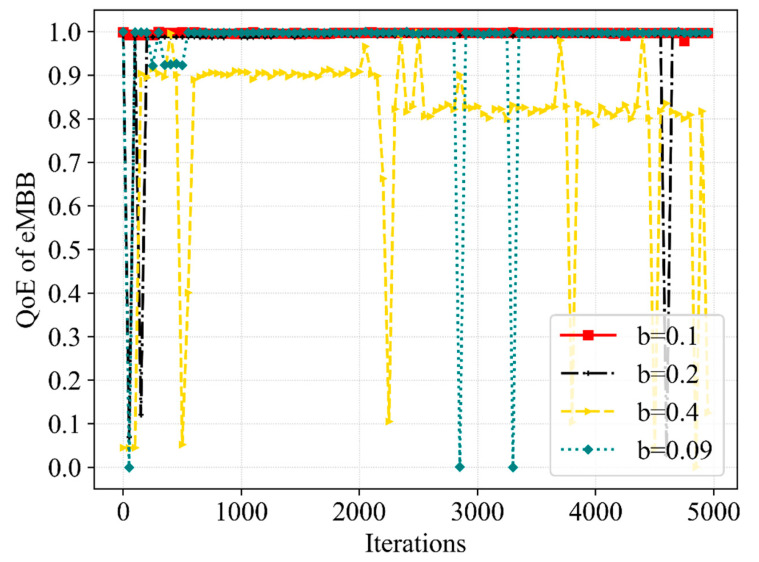
A comparative result of QoE for eMBB service at various b.

**Figure 12:**
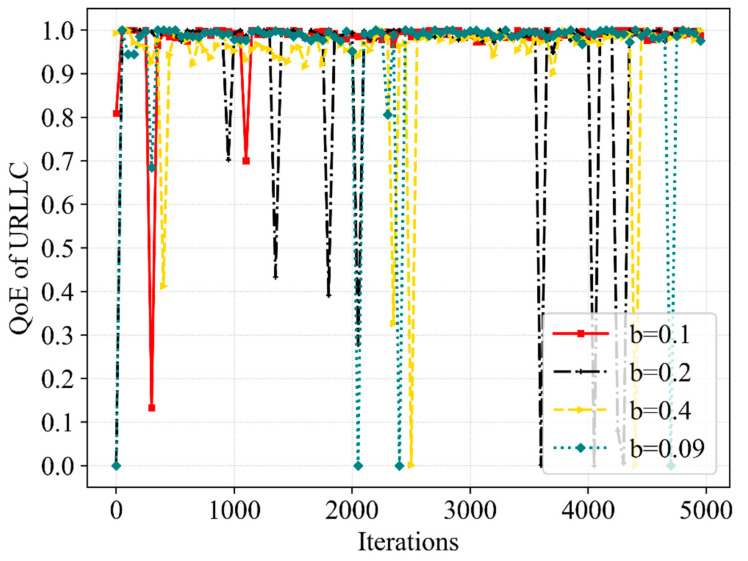
A comparative result of QoE for URLLC service at various b.

Figure 11 and Figure 12 compare the QoE tendency of eMBB and URLLC in the different cases in which the b is 0.1 MHz, 0.2 MHz and 0.4 MHz. It shows that the QoE converges almost to the optimum at the condition that b is 0.1 MHz, but the convergence is slightly worse when b is 0.09 MHz and 0.2 MHz. It is worth noting that when b is 0.4 MHz, the QoE of eMBB struggles to satisfy the demand, and the QoE of URLLC also has worse stability. Although we set up a mechanism to only schedule bandwidth beyond the minimum required to satisfy eMBB when performing bandwidth resource scheduling, the QoE of eMBB is severely affected by the large number of bandwidth resources being scheduled each time.

**Figure 13:**
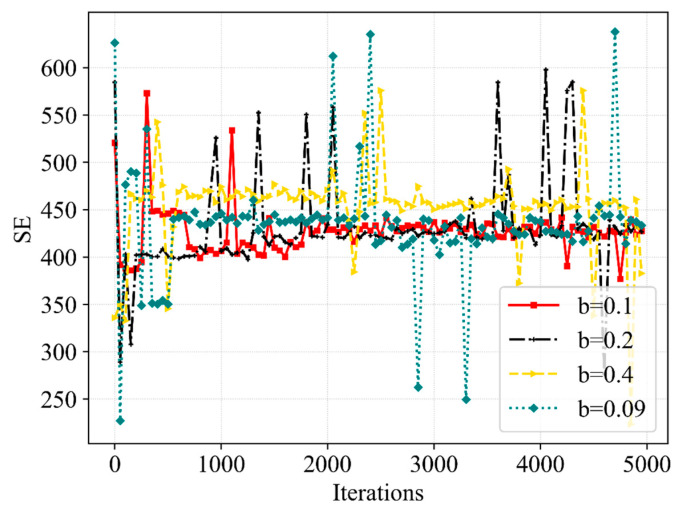
A comparative result of SE at various b.

**Figure 14:**
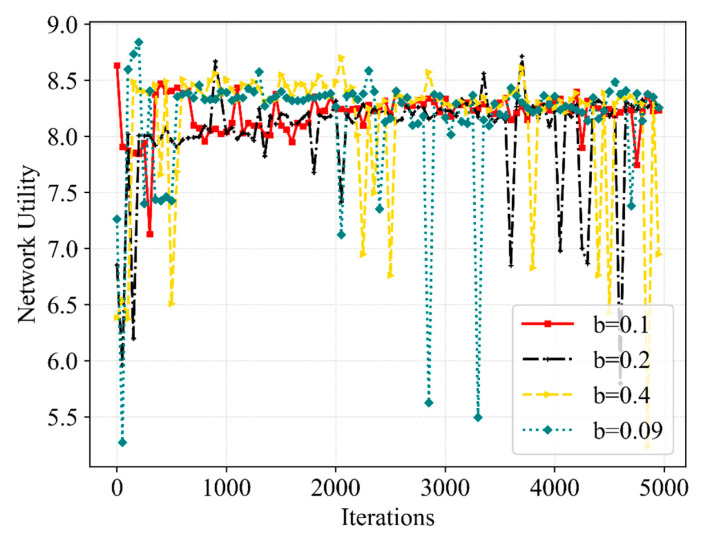
A comparative result of network utility at various b.

Figure 13 and Figure 14 further compare the performance of the SE and network utility in the different cases. From the perspective of SE, the convergence of the Dueling DQN is significantly superior to other cases when b is 0.1 MHz. Although the SE converges to a higher value at b of 0.4 MHz, the QoE fails to satisfy the requirements at this moment. Meanwhile, the scheduling of bandwidth resources is more flexible when b is 0.09 MHz, but the stability of SE is not improved. From the perspective of network utility, among the mentioned b, the convergence speed is the slowest when b is 0.09 MHz and shows a slight difference in other cases. More bandwidth resources will be scheduled without interrupting eMBB user services when we slice the bandwidth more finely. Hence, the delicate b can better improve the QoE of URLLC without reducing the QoE of eMBB and can obtain better stability of SE and network utility, due to the increased flexibility in bandwidth resource allocation and scheduling. Moreover, the b we set has a great impact on the action space; the excessively delicate b can also make the action space too large, leading to a more complex resource allocation process, which influences the effectiveness of the latency time of service and the efficiency of bandwidth allocation and scheduling. The choice of 0.1 MHz for b in this paper makes the proposed algorithm perform better when solving the resource allocation and scheduling problem.

After the display and analysis of the above simulation results, the following conclusions can be obtained. Dueling DQN and the resource scheduling mechanism contribute to the stability of QoE and the improvement of SE and network utility. Moreover, the selection of b obtains the appropriate size of action space and improves the flexibility of resource allocation, and the use of the reward-clipping mechanism enables the proposed algorithm to obtain a more stable performance.

## 5. Conclusions

In this paper, the optimization problem of resource allocation and scheduling in heterogeneous integrated networks is proposed, and the dueling deep Q network (Dueling DQN)-based algorithm for eMBB and URLLC hybrid services is proposed to solve this problem. To prioritize URLLC service requirements, a resource scheduling mechanism between eMBB and URLLC services is proposed. To solve this formulated optimization problem with non-convex properties in relation to the allocation and scheduling of bandwidth resources, an iterative Dueling DQN-based algorithm is proposed. In addition, to enhance the training stability of Dueling DQN, the reward-clipping mechanism is adopted. Moreover, to increase flexibility in resource allocation, a suitable bandwidth allocation resolution (b) is chosen. We verify through simulations that the algorithm based on Dueling DQN for resource allocation and scheduling has excellent performances for quality of experience (QoE), spectrum efficiency (SE) and network utility. We also verify that the Dueling DQN-based algorithm is much better suited to tackling this problem than the Q-learning, DQN and Double DQN, and the resource scheduling mechanism significantly increases the stability of performances.

## Figures and Tables

**Table 1:** Simulation Parameters.

	eMBB	URLLC
Channel	Rayleigh fading	
Scheduling	1 s (2000 scheduling slots)	
Noise Spectral Density (σ)	−174 dBm/Hz	
Total Bandwidth (B)	20 MHz	
Bandwidth Allocation Resolution (b)	0.1 MHz	
Number of Users (M)	500	
	300	200
Minimum Rate Constraint (r)	100 Mbps	10 Mbps
Maximum Delay Constraints (t)	10 ms	1 ms
Distribution of Users	Truncated Pareto [=Exponential Parameter: 1.2, Average: 6 ms, Maximum: 12.5 ms]	Constant: 0.3 MByte
Distribution of pkt	Truncated Pareto [Exponential Parameter: 1.2, Average: 100 Byte, Maximum: 250 Byte]	Exponential [Mean: 180 ms]

## Data Availability

Data sharing not applicable.
